# Three-Dimensional Pharyngeal Airway Space Changes Following Isolated Mandibular Advancement Surgery in 120 Patients: A 1-Year Follow-up Study

**DOI:** 10.3390/jimaging8040082

**Published:** 2022-03-22

**Authors:** Sohaib Shujaat, Eman Shaheen, Marryam Riaz, Constantinus Politis, Reinhilde Jacobs

**Affiliations:** 1 OMFS IMPATH Research Group, Department of Imaging & Pathology, Faculty of Medicine, KU Leuven & Oral and Maxillofacial Surgery, University Hospitals Leuven, 3000 Leuven, Belgium; eman.shaheen@uzleuven.be (E.S.); stan@politis.be (C.P.); reinhilde.jacobs@kuleuven.be (R.J.); 2 Department of Physiology, Azra Naheed Dental College, Superior University, Lahore 54600, Pakistan; drmarryamphysiology@gmail.com; 3 Department of Dental Medicine, Karolinska Institutet, 17177 Stockholm, Sweden

**Keywords:** orthognathic surgical procedures, 3D imaging, cone-beam computed tomography, pharynx, follow-up studies

## Abstract

Lack of evidence exists related to the three-dimensional (3D) pharyngeal airway space (PAS) changes at follow-up after isolated bilateral sagittal split osteotomy (BSSO) advancement surgery. The present study assessed the 3D PAS changes following isolated mandibular advancement at a follow-up period of 1 year. A total of 120 patients (40 males, 80 females, mean age: 26.0 ± 12.2) who underwent BSSO advancement surgery were recruited. Cone-beam computed tomography (CBCT) scans were acquired preoperatively (T0), immediately following surgery (T1), and at 1 year of follow-up (T2). The volume, surface area, and minimal cross-sectional area (mCSA) of the airway were assessed. The total airway showed a 38% increase in volume and 13% increase in surface area from T0 to T1, where the oropharyngeal region showed the maximum immediate change. At T1–T2 follow-up, both volumetric and surface area showed a relapse of less than 7% for all sub-regions. The mCSA showed a significant increase of 71% from T0 to T1 (*p* < 0.0001), whereas a non-significant relapse was observed at T1–T2 (*p* = 0.1252). The PAS remained stable at a follow-up period of 1 year. In conclusion, BSSO advancement surgery could be regarded as a stable procedure for widening of the PAS with maintenance of positive space at follow-up.

## 1. Introduction

Dentofacial deformity in skeletal class II patients is most commonly characterized by mandibular retrognathism [1]. One of the most highly stable, predictable, and widely accepted surgical techniques for correcting the skeletal, soft tissue, and dental discrepancies in such patients involves bilateral sagittal split osteotomy (BSSO) advancement surgery [2]. Advancement of the mandible not only protrudes the mandible into a desirable position but also influences the pharyngeal airway space (PAS) by altering the hyoid bone position, the supra- and infra-hyoid, and the base of the tongue musculature [3]. These anatomical changes have the tendency to increase the airway volume and dimensions by repositioning of the pharyngeal soft tissue anteriorly [4]. This increase in PAS not only improves the airway patency in obstructive sleep apnea (OSA) patients but it also improves the respiratory status and sleep quality in non-OSA patients without any breathing or respiratory disorders [5]. 

Since the mid-1980s, numerous studies have been carried out assessing airway changes in patients undergoing mandibular advancement surgery [6,7]. With the technological advancements, three-dimensional (3D) volumetric assessment of the PAS has become an objective standard method for assessing the airway compared to its conventional 2D counterparts such as orthopantomogram and lateral cephalogram [8]. The 2D PAS changes following bimaxillary, single jaw advancement, and/or setback surgeries have been extensively studied. However, there are only a few studies related to the 3D PAS changes in skeletal class II patients following isolated mandibular advancement and how the airway changes at follow-up [9,10,11,12]. Apart from volumetric changes following mandibular advancement, another important parameter known as the most restricted or minimum cross-sectional area (mCSA) of the PAS has been reported in literature [13]. The mCSA predicts the collapsibility of PAS and is an important parameter for assessing the airway resistance [14]. Although numerous studies have assessed mCSA in patients requiring orthognathic surgery [13], few studies are available focusing on the mCSA changes at follow-up utilizing CBCT in patients treated with isolated BSSO advancement surgery [9].

The main limitation of the aforementioned studies has either been related to their small sample size [15,16] or short-term follow-up with a maximum duration of 6 months [12]. To our knowledge, currently no evidence exists evaluating 3D airway changes in a large group of patients with a follow-up of 1 year or more in patients undergoing isolated mandibular advancement. Therefore, the current study was conducted to address two aims. The first aim was to three-dimensionally assess the volumetric and surface area changes of the airway following isolated mandibular advancement at a follow-up period of 1 year. The second aim investigated the changes in collapsibility of the airway by assessing the mCSA.

## 2. Materials and Methods

A total of 120 patients were recruited, consisting of 40 male and 80 female patients having a mean age of 26.0 ± 12.2 and a follow-up period of 12.0 ± 2.6 months. Data were collected from a period of August 2014 through March 2020. Patients who underwent isolated mandibular advancement surgery without genioplasty and had a craniocervical angle (N-S-Ba) of less than 5° for overcoming the variation in head position [16] were included in the study. Exclusion criteria involved patients with craniofacial anomalies, syndromic disorders, OSA, previous history of trauma, or any other orthognathic surgery procedure such as Le Fort I or genioplasty. All surgeries were performed by the same surgical team and involved BSSO advancement based on Hunsuck/Epker modification with transoral rigid internal fixation of the osteotomized segment with two miniplates and monocortical screws on each side [17,18,19,20]. 

Cone-beam computed tomography (CBCT) scans of the patients were acquired preoperatively (T0), immediately following surgery at an interval of 1–6 weeks (T1), and at 1 year of follow-up (T2). All scans were acquired using a standardized scanning protocol suggested by Stratis et al. [21], and patients were in a relaxed and upright position with the Frankfort Horizontal (FH) plane parallel to the floor. Two CBCT devices were utilized for acquiring the scans, Planmeca Promax 3D Max (Planmeca, Helsinki, Finland) and Newtom VGi-evo (Newtom, Verona, Italy). The scanning parameters were set at a 230 × 260 to 240 × 190 mm^2^ field of view, 96–110 kV, and a slice thickness of 0.3–0.6 mm. Following CBCT acquisition, scans were exported in DICOM (Digital Imaging and Communications in Medicine) format. The T0 images were re-oriented and adjusted to the FH plane where required.

**Figure 1:**
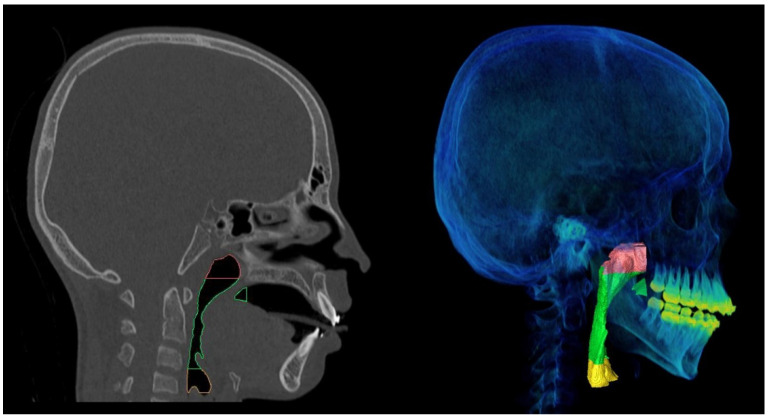
Pharyngeal airway space sub-regions division with posterior nasal spine (PNS) as the anterior vertical limit and pharyngeal soft tissue contour as the posterior limit. Red color indicates nasopharyngeal region extending from root of clivus superiorly to PNS inferiorly. Green color indicates oropharyngeal area extending from PNS superiorly to anterior–inferior point of the body of third cervical vertebrae (C3ai) inferiorly. Yellow color indicates hypopharyngeal region extending from C3ai superiorly to anterior–inferior point of the body of fourth cervical vertebrae inferiorly (C4ai).

Voxel-based registration was applied utilizing the anterior cranial base for superimposition of T1 and T2 scans onto the T0 scan using Amira software (version 2019.3, Thermo Fischer Scientific, Merignac, France). Following image registration, all data were imported to ProPlan CMF 3.0 (Materialise, Leuven, Belgium), where segmentation of airway was performed with the initial threshold setting between −1024 to −500 Hounsfield units (HU). Additionally, manual adjustment was performed in cases where a proper depiction of the airway was not observed. Following segmentation, planes were reconstructed for dividing the PAS into the following anatomical regions: nasopharynx, oropharynx, hypopharynx, and complete PAS. The division of sub-regions was performed based on previously validated anatomical and technical limits proposed by Guijarro-Martínez and Swennen (Figure 1) [22]. The volume and surface area of the segmented structures were then calculated. 

**Figure 2:**
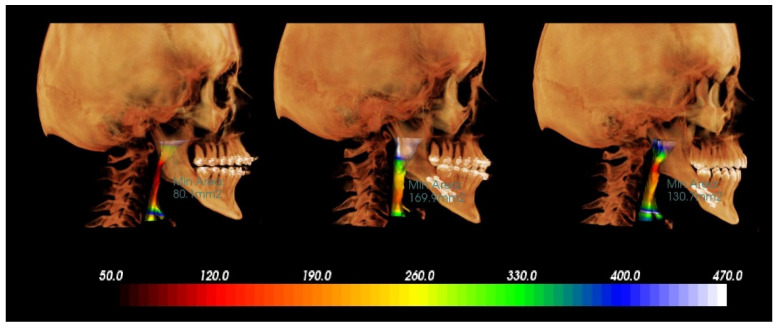
Illustration of minimum cross-sectional area changes following mandibular advancement surgery, left side: before surgery, middle: 2 weeks following surgery, right side: 1 year after surgery.

Thereafter, the DICOM data set was imported to InVivo Anatomage software (version 5.4, Anatomage, San Jose, CA, USA) for determining the mCSA of the complete airway. It was defined as the minimal cross-sectional area along the airway axially extending from the posterior nasal spine superiorly until the anterior–inferior point of the body of the fourth cervical vertebrae inferiorly. The software automatically detected and calculated the mCSA (Figure 2). Validity and reliability of the software for calculating mCSA has been previously reported [23].

## 3. Statistical Analysis

Data were analyzed with MedCalc Statistical Software version 19.2 (MedCalc Software Ltd., Ostend, Belgium). Descriptive statistics including percentage, mean, and standard deviation were calculated for all the data. Normality of data was assessed with the Shapiro–Wilk test. For normally distributed data, a t-test was utilized to determine the change in PAS parameters from T0 to T1 and T1 to T2. A Wilcoxon signed-rank test was applied for data with non-parametric distribution. A Spearman correlation coefficient was calculated for assessing the relationship between the amount of advancement and mCSA change immediately after surgery and at follow-up. Statistical significance was set at 0.05 for all parameters.

## 4. Results

Patients underwent a mean mandibular body advancement of 5.7 ± 2.3 mm anteriorly and a relapse of −1.2 ± 1.2 mm posteriorly. No significant difference was observed in relation to the cranio-cervical angle (*p* > 0.062), thereby confirming the stability of head position. 

Table 1 describes the mean and percentage of change for volume, surface area, and mCSA at T0–T1 and T1–T2 time intervals. The total airway showed a 38% increase in total airway volume and 13% increase in surface area from T0 to T1. The oropharyngeal region showed the maximum change in airway volume and surface area, followed by the hypopharynx and nasopharynx. The surgery immediately led to a significant increase in total airway volume and surface area (*p* < 0.0001) from T0 to T1. When divided into sub-regions, most of the significant volumetric changes occurred in the oropharyngeal region followed by the nasopharynx (*p* < 0.0001). No significant volumetric changes were seen in the hypopharyngeal region immediately following surgery (*p* = 0.948).

**Figure 3:**
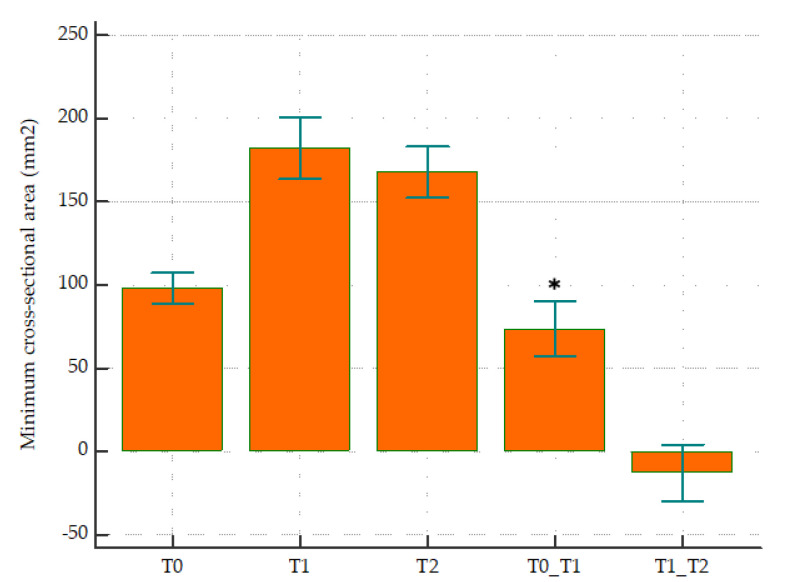
Minimum cross-sectional area (mm^2^) at (T0) before surgery, (T1) immediately after surgery, (T2) 1-year follow-up, T0 to T1, and T1 to T2 time intervals. * represents statistical significance (*p* < 0.05).

At T1–T2 follow-up, both volumetric and surface area showed a relapse of less than 7% for all sub-regions, where the total airway volume decreased by 5% and the surface area increased by 3%. Among the volumetric measurements, only the total airway volume (*p* = 0.004) and oropharyngeal sub-region (*p* = 0.004) showed a significant decrease. No significant changes in surface area were observed at follow-up, with both total airway and all sub-regions showing an increase in surface area. According to Table 1 and Figure 3, the mCSA of the airway showed a significant increase of 71% from T0 to T1 (*p* < 0.0001), whereas a non-significant relapse in the opposite direction was observed at T1–T2 (−15%, *p* = 0.125).

Supplementary Materials Table S1 demonstrates the gender-based changes in the airway. Based on the gender of the patient, approximately similar changes were observed at T0–T1 and T1–T2 time intervals. 

**Figure 4:**
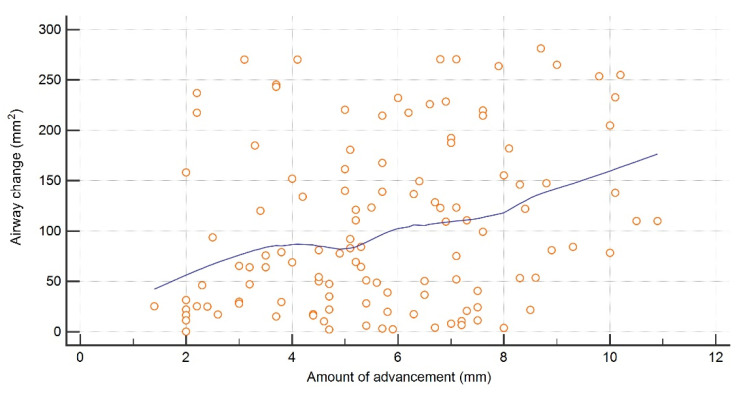
Correlation between amount of mandibular advancement and minimum cross-sectional area.

The immediate changes in airway mCSA showed a significantly weak correlation with the amount of advancement (r = 0.25, *p* < 0.0049) (Figure 4). Both the total PAS and anatomical sub-regions showed a negligible to weak correlation with the amount of movement and relapse, where all the values were <0.39 and gender had no impact on the correlation (Table 2). Furthermore, no significant age- and gender-related differences were observed for all the parameters.

## 5. Discussion

The present study was conducted to address volumetric, surface area, and mCSA changes immediately after surgery and at a 1-year follow-up in a large group of patients. Our findings suggested a significant increase in the total PAS and mCSA immediately following surgery. The most likely explanation for this change could be related to the anatomical changes achieved by the BSSO advancement, where the most prominent change includes the elevation of the hyoid bone during surgery with supero-anterior movement due to the conjoint response of supra- and infra-hyoid muscles and change in tongue position [24]. 

The oro- and hypopharyngeal region showed the highest change in volume immediately after surgery. This could be attributed to the stretching of the genioglossus and geniohyoid muscles, which originate from the mental spine and are responsible for protruding the tongue and hyoid bone anteriorly [25]. These muscles increase the soft tissue tension of the oro/hypopharyngeal region, thereby leading to expansion of the PAS. Additionally, mandibular advancement also puts tension on the palatoglossus muscle, which arises from the soft palate and attaches to the side of the tongue [4,26], thereby resulting in a further change of the oropharyngeal region. Mandibular advancement further influences the pharyngeal dilators by changing their tone. When combined with hyoid bone movement and stretching of the associated muscle attachments, this might have led to increased hypopharyngeal patency and resistance [25]. At the same instance, the nasopharynx also showed a significant increase in volume. It seems surprising that mandibular advancement led to the increase in the nasopharyngeal region even without any maxillary intervention. A possible explanation for this could be related to the tension transmitted to the soft palate and posterior wall of the pharynx through the palatopharyngeal muscles [5,27]. However, changes in the nasopharyngeal area were minimal, potentially related to the dorsocranial anatomy limiting nasopharyngeal movement [12]. 

Additionally, a negligible to weak correlation existed between the PAS changes and skeletal changes at both immediate and follow-up time points, thereby also confirming that the skeletal relapse had a minimal influence on the PAS volume and dimensions. As no studies were found correlating skeletal movement and relapse with PAS changes in isolated mandibular advancement cases at follow-up, a comparison with similar literature was not possible. Nevertheless, AlSaty et al. also did not find any correlation between skeletal relapse and change in airway space following maxillomandibular advancement with and without genial tubercle advancement [28].

The significant immediate increase in the airway dimensions and mCSA immediately after surgery was consistent with other studies [4,12,15,29]. A variation in the percentage of change existed when compared with these studies, which could have resulted due to the mean amount of movement and heterogeneity of data based on the sample size and landmarks utilized for segmenting airway sub-regions. Additionally, based on the correlation analysis, some patients had a little while others had more change in mCSA with the same amount of advancement immediately after surgery, thereby confirming that at an individual patient level change in mCSA cannot be predicted based on the amount of advancement. These inconsistencies in mCSA could have resulted due to the breathing movements, tongue positioning, post-operative edema, and variability in soft tissue compensation following new skeletal positioning [4,7,12]. We also believe that the amount of rotational skeletal movement and wax bite thickness could have also contributed to the mCSA variability among patients requiring the same amount of advancement.

At follow-up, a significant decrease in the total airway volume was observed, where the oropharyngeal region showed maximal change. Even though mandibular advancement has been known to be a stable procedure, skeletal relapse of the distal segment is still observed in a posterior direction at follow-up. This posterior relapse is associated with recoiling of the hyoid musculature, which exerts force in a posterior direction, thereby acting as a precipitating factor for causing the oropharyngeal airway to relapse [30]. Our findings were consistent with another study that showed a decrease in total airway volume of approximately 4% at follow-up, which was comparable to the 5% change seen in this study [31]. Volumetric and surface relapse of the total airway and its sub-regions were within the range of −2% to 6%, which confirmed that the airway remained clinically stable at follow-up. Although certain sub-regions showed statistically significant relapse at follow-up, the overall changes at a 1-year time point were clinically insignificant.

Instead of assessing the constriction in each segment separately, mCSA was assessed for the complete oro/hypopharyngeal airway. We believe that by doing so it provides more clinically relevant information about how the constriction changes for the complete airway instead of focusing on the segments separately for mandibular advancement surgery [8]. A 71% increase in mCSA was observed immediately following surgery, which then showed a non-significant relapse of −15% at follow-up. This again confirms that mandibular advancement surgery influences the complete palatoglossohyoid muscle and ligament system, thereby not just increasing the volume but also the cross-sectional area [12]. The increase in mCSA has been associated with the tension produced by stretching of the suprahyoid and velopharyngeal muscles [32,33]. These muscles intend to go back to their original position, which could explain the decrease in mCSA at follow-up. Nevertheless, all parameters showed clinically insignificant changes at follow-up.

The study had certain limitations. Firstly, the breathing movements during the CBCT scan acquisition were not controlled, which could have led to bias within our findings. However, the large sample size could have overcome these small inconsistencies. Secondly, the tongue movement was also not controlled at all the time points. In the midst of these limitations, we believe that our study provides a clinically relevant update on airway changes in patients undergoing isolated mandibular advancement. Further comparative studies should be conducted to assess PAS follow-up changes following single- and double-jaw surgeries and to assess whether airway changes differ between OSA and non-OSA patients.

## 6. Conclusions

Isolated BSSO advancement surgery led to a significant, immediate increase in the total airway volume, surface area, and minimum constriction area. All these changes remained stable at a 1-year follow-up. Based on our findings, BSSO advancement surgery could be regarded as a stable procedure for widening of the PAS with maintenance of positive space at follow-up.

## Figures and Tables

**Table 1:** Mean ± standard deviation and relative change (RC %) of airway volume, surface area, and minimum cross-sectional area (mCSA).

PAS	T0 (Mean ± SD)	T1 (Mean ± SD)	T2 (Mean ± SD)	RC % (T0–T1)	RC % (T1–T2)
Volume (mm^3^)					
TA	21,194.14 ± 5113.33	28,617.26 ± 8032.89	26,556.54 ± 8055.54	**38%**	**−5%**
NP	5437.92 ± 2175.23	5919.18 ± 2101.36	5812.14 ± 2249.14	**14%**	−2%
OP	14,773.96 ± 4813.27	21,908.69 ± 6828.23	20,136.73 ± 6680.50	**59%**	**−4%**
HP	3243.95 ± 1330.66	3259.58 ± 1624.11	3529.96 ± 2024.87	21%	2%
Surface area (mm^2^)					
TA	10,869.39 ± 2148.63	12,095.80 ± 2476.5	12,273.99 ± 2884.84	**13%**	3%
NP	3081.18 ± 821.31	3158.71 ± 804.38	3249.71 ± 868.29	5%	4%
OP	6771.97 ± 1713.87	7939.60 ± 1881.65	8008.57 ± 2010.05	**22%**	4%
HP	1925.02 ± 657.01	1875.73 ± 674.8	1947.94 ± 763.86	8%	6%
mCSA (mm^2^)					
	98.07 ± 47.59	182.29 ± 93.90	167.48 ± 78.96	**71%**	−15%

PAS: pharyngeal airway space; TA: total airway; NP: nasopharynx; OP: oropharynx; HP: hypopharynx; T0: before surgery; T1: immediately after surgery; T2: 1-year follow-up; SD: standard deviation; %: percentage; Sign: significance; −ve percentage indicates decrease; +ve percentage indicates increase; bold value indicates statistical significance.

**Table 2:** Overall and gender-based correlation between pharyngeal airway space and skeletal movement and relapse.

PAS	Movement Correlation			Relapse Correlation		
	Volume					
	T0-T1			T1–T2		
	Overall	Male	Female	Overall	Male	Female
**TA**	0.16	0.26	0.11	0.16	0.11	−0.06
**NP**	**−0.19**	−0.08	**−0.23**	**−0.19**	0.17	0.03
**OP**	**0.32**	0.24	**0.33**	**0.32**	−0.09	0.09
**HP**	0.06	−0.13	0.14	0.06	0.16	0.21
	Surface area					
	T0-T1			T1–T2		
	Overall	Male	Female	Overall	Male	Female
**TA**	0.15	0.16	0.14	0.00	0.06	−0.07
**NP**	−0.23	−0.31	−0.18	−0.04	0.09	−0.14
**OP**	0.29	**0.33**	**0.25**	−0.09	−0.21	−0.05
**HP**	0.05	−0.04	0.06	0.15	0.18	0.12
	Minimum cross-sectional area					
	T0-T1			T1–T2		
	Overall	Male	Female	Overall	Male	Female
	**0.25**	**0.38**	**0.26**	0.13	**0.34**	0.00

PAS: pharyngeal airway space; TA: total airway; NP: nasopharynx; OP: oropharynx; HP: hypopharynx; T0: before surgery; T1: immediately after surgery; T2: 1-year follow-up; bold value indicates statistical significance.

## Data Availability

The data presented in this study are available on request from the corresponding author.

## Supplementary Materials

The following supporting information can be downloaded at https://www.mdpi.com/article/10.3390/jimaging8040082/s1. Table S1: Gender-based mean ± standard deviation and relative change (RC %) of airway volume, surface area, and minimum cross-sectional area (mCSA).
