# Supplementary material for: Three-Dimensional Pharyngeal Airway Space Changes Following Isolated Mandibular Advancement Surgery in 120 Patients: A 1-Year Follow-up Study
Source: J Imaging. 2022 Mar 22;8(4):82. doi: 10.3390/jimaging8040082 (PMC9029548; doi:10.3390/jimaging8040082)
Supplement: Supplementary file 1 [file jimaging-08-00082-s001.zip › jimaging-1563245-supplementary.pdf]

Supplementary Materials: Three-dimensional pharyngeal airway space changes following isolated mandibular advancement surgery in 120 patients: a 1-year follow-up study

Sohaib Shujaat , Eman Shaheen , Marryam Riaz , Constantinus Politis and Reinhilde Jacobs

Table S1. Gender-based mean ± standard deviation and relative change (RC %) of airway volume, surface area, and minimum cross-sectional area (mCSA).

PAS	Gender	T0 (Mean ± SD)	T1 (Mean ± SD)	T2 (Mean ± SD)	RC % (T0-T1)	RC % (T1-T2)
			Volume (mm³)			
TA	M	21,973.78 ± 4441.02	31,606.99 ± 7965.66	29,133.66 ± 9980.53	**44%**	**−7%**
	F	20,895.11 ± 5347.06	27,470.51 ± 7811.37	25,568.06 ± 7014.25	**35%**	**−4%**
NP	M	5988.74 ± 2258.74	6551.51 ± 2102.76	6440.40 ± 2316.51	**18%**	−2%
	F	5179.51 ± 2099.48	5622.53 ± 2046.75	5517.41 ± 2168.81	13%	−2%
OP	M	16,136.11 ± 4763.76	25,551.34 ± 6236.42	23,347.08 ± 7151.39	**68%**	**−5%**
	F	14,192.77 ± 4747.07	20,354.49 ± 6504.43	18,766.99 ± 6012.10	**56%**	**−3%**
HP	M	3826.93 ± 1343.77	3846.69 ± 1632.21	4223.54 ± 2310.73	15%	5%
	F	2983.14 ± 1255.92	2996.92 ± 1571.11	3219.68 ± 1831.68	24%	2%
			Surface area (mm²)			
TA	M	11,378.27 ± 2057.48	13,018.20 ± 2151.41	13,455.60 ± 3347.67	**18%**	3%
	F	10,650.78 ± 2173.96	11,674.80 ± 2495.98	11,821.66 ± 2544.49	**11%**	3%
NP	M	3250.17 ± 902.14	3327.33 ± 769.69	3497.95 ± 909.77	8%	6%
	F	3000.90 ± 773.03	3078.62 ± 812.79	3131.79 ± 827.77	4%	2%
OP	M	7178.99 ± 1639.53	8709.85 ± 1627.97	8857.10 ± 2125.02	**27%**	5%
	F	6581.34 ± 1724.72	7578.84 ± 1893.05	7611.16 ± 1836.08	**19%**	4%
HP	M	2234.68 ± 707.89	2237.08 ± 695.51	2356.63 ± 922.91	8%	11%
	F	1778.34 ± 585.40	1704.57 ± 600.84	1754.35 ± 596.86	8%	13%
			mCSA (mm²)			
	M	101.88 ± 45.50	196.74 ± 99.34	174.72 ± 80.52	**61%**	−11%
	F	96.31 ± 48.74	175.58 ± 91.21	164.11 ± 78.57	**75%**	−15%

PAS: pharyngeal airway space; TA: total airway; NP: nasopharynx; OP: oropharynx; HP: hypopharynx; T0: before surgery; T1: immediately after surgery; T2: 1-year follow-up; SD: standard deviation; %: percentage; Sign: significance; -ve percentage indicates decrease; +ve percentage indicates increase; bold value indicates statistical significance.
